# Identification of Glycoside Transporters From the Human Gut Microbiome

**DOI:** 10.3389/fmicb.2022.816462

**Published:** 2022-03-25

**Authors:** Zhi Wang, Alexandra S. Tauzin, Elisabeth Laville, Gabrielle Potocki-Veronese

**Affiliations:** TBI, CNRS, INRA, INSAT, Université de Toulouse, Toulouse, France

**Keywords:** microbiome, oligosaccharides, CAZymes, functional metagenomics, transporters

## Abstract

Transport is a crucial step in the metabolism of glycosides by bacteria, which is itself key for microbiota function and equilibrium. However, most transport proteins are function-unknown or only predicted, limiting our understanding of how bacteria utilize glycosides. Here, we present an activity-based screening method to identify functional glycoside transporters from microbiomes. The method is based on the co-expression in *Escherichia coli* of genes encoding transporters and carbohydrate-active enzymes (CAZymes) from metagenomic polysaccharide utilization loci (PULs) cloned in fosmids. To establish the proof of concept of the methodology, we used two different metagenomic libraries derived from human gut microbiota to select 18 *E. coli* clones whose metagenomic sequence contained at least one putative glycoside transporter and one functional CAZyme, identified by screening for various glycoside-hydrolase activities. Growth tests were performed on plant-derived glycosides, which are the target substrates of the CAZymes identified in each PUL. This led to the identification of 10 clones that are able to utilize oligosaccharides as sole carbon sources, thanks to the production of transporters from the PTS, ABC, MFS, and SusCD families. Six of the 10 hit clones contain only one transporter, providing direct experimental evidence that these transporters are functional. In the six cases where two transporters are present in the sequence of a clone, the transporters’ function can be predicted from the flanking CAZymes or from similarity with transporters characterized previously, which facilitates further functional characterization. The results expand the understanding of how glycosides are selectively metabolized by bacteria and offers a new approach to screening for glycoside-transporter specificity toward oligosaccharides with defined structures.

## Introduction

Bacteria have evolved diverse metabolic lifestyles and strategies to harvest energy from glycosides (Ndeh and Gilbert, 2018). According to the current paradigm, utilization of glycosides in the *Bacteroidetes*, a dominant phylum of the mammalian gut microbiota, is usually orchestrated by polysaccharide utilization loci (PULs), which encode carbohydrate-active enzymes (CAZymes) associated with SusCD-like transport systems, and sometimes also transporters of the ATP-binding cassette (ABC) or Major Facilitator Superfamily (MFS) families (Terrapon et al., 2018). In another dominant phylum of the mammalian gut microbiota, the *Firmicutes*, glycosides are metabolized by systems called gpPULs (Gram-positive PULs), which are analogous to canonical PUL systems. Rather than encoding TonB-dependent receptors, though, they code for a range of other transporters, including ABC, MFS, and phosphotransferase systems (PTS) (Sheridan et al., 2016). Depending on their degree of polymerization (DP), polysaccharides are partially degraded by cell surface-associated CAZymes to oligomers, which are then imported into the cell periplasm (for Gram-negative bacteria) or cell cytoplasm (Gram-positive) through transporters. In the cell periplasm or the cytoplasm, oligosaccharides are processed by intracellular CAZymes to monosaccharides that are ultimately assimilated into the central metabolism of the bacteria. Glycoside transporters are therefore critical for glycoside utilization by bacteria, and thus linked to their ability to settle in nutritional niches (Grondin et al., 2017).

Understanding how bacteria recognize and transport glycosides is key to understanding gut microbiota function and thus to improving health and preventing disease (Marques et al., 2018). For instance, ABC transporters are indeed likely to provide *Bifidobacteria* with a significant advantage in capturing oligosaccharides via their extracellular lipid-anchored solute-binding proteins (SBPs) in the competitive human gut niche (Theilmann et al., 2019). Furthermore, the presence of glycoside-specific PTS or MFS transporters in pathogenic bacteria, including *Escherichia coli* BEN2908 (Schouler et al., 2009), was found to be correlated with the colonization and virulence of pathogens in the gut. In addition, for biotechnological applications, better knowledge of glycoside uptake will enable the development of microbial cell factories and microbial consortia with the ability to efficiently convert non-refined biomass to high-value products (Hara et al., 2017).

Despite the importance of bacterial glycoside transporters for health and biotechnology, the rate of functional characterization of transporters is much slower than for CAZymes (Lombard et al., 2014). One of the major challenges lies in finding the correct substrates among the extremely wide diversity of oligosaccharides (estimated at as many as a few thousand Lapébie et al., 2019) for each transporter. CAZymes are classified into families based on sequence and mechanistic similarities in the CAZy database, and their function can be predicted based on homology with the numerous biochemically characterized members of the same family (Lombard et al., 2014). For transporters, the classification of membrane transport proteins based on the Transporter Classification (TC) system, which is accessible in the Transporter Classification Database (TCDB), is helpful too, since it incorporates both functional and phylogenetic information (Saier et al., 2016). However, predicting transporter substrate specificity is extremely difficult due to their low sequence similarity and to the fact that the vast majority of annotated glycoside-specific transporters lack experimental validation (Genee et al., 2016; Elbourne et al., 2017). Indeed, there are several challenges associated with the experimental investigation of glycoside transporter function. The usual method of identifying transporter specificity is based on monitoring the accumulation of radiolabeled compounds in whole cells, membrane vesicles or proteoliposomes (Blanvillain et al., 2007; Cao et al., 2011; McCoy et al., 2016). However, these assays can easily fail when the heterologous expressed transmembrane transport proteins are inactive due to targeting, insertion or folding issues, or when the transporters are insoluble or unstable in purification or refolding procedures (Majd et al., 2018). In addition, some substrates are not available in radioactive form or are prohibitively expensive, thus precluding large-scale identification trials. Transporter specificity can also be validated in native strains by transcriptional analysis after cultivating the target strains on glycosides (Martens et al., 2011), via complementation studies (Joglekar et al., 2018) or by analysis of the phenotype of knock-out mutants (Linke et al., 2013). Nevertheless, the success of these approaches is still limited because of (i) the cost of omics studies, which is prohibitive if one wants to screen the metabolization potential of a large panel of oligosaccharides with defined structures, (ii) the difficulty of genetic manipulation with some species, (iii) the fact that functional redundancy often occurs in bacteria (Jensen et al., 2002; Maharjan et al., 2007), (iv) the fact that most bacteria in natural ecosystems are uncultured (Steen et al., 2019). There is thus a need to develop new methods for rapid identification and validation of glycoside-transporter functions.

Here, we present a functional metagenomic approach for rapid identification of glycoside-transporters from uncultured human gut bacteria, which do not require radiolabeled substrates. The specificity of the transport systems was screened via growth assays in micro-plates of fosmid *E. coli* clones containing metagenomic PULs. These growth assays enabled us to rapidly identify several types of glycoside transporters that are functional in *E. coli*, revealing novel functions of uncultured bacteria of various taxonomical origins.

## Results

### Prediction of Potential Glycoside Transporters From Metagenomic Clones

Forty-three metagenomic clones isolated during previous functional screening campaigns of the human gut microbiome for their ability to hydrolyze plant polysaccharides and oligosaccharides (Tasse et al., 2010; Cecchini et al., 2013), and subsequently sequenced, were selected to investigate their ability to transport oligosaccharides. Each of these metagenomic clones comprises within its 30–40 kb DNA insert at least one gene encoding a CAZyme responsible for the glycoside-hydrolase activity being screened for. Of the 43 clones, 26 produced at least one endo-acting glycoside-hydrolase activity. They derived from a 156,000-clone metagenomic library constructed from the feces of a healthy adult with a fiber-rich diet (F library), screened for the degradation of plant polysaccharides via chromogenic assays (Tasse et al., 2010). Seventeen other clones produced at least one glycosidase activity. These were identified from 20,000 clones chosen at random from the F library, and from another 20,000-clone library constructed from the ileum mucosal microbiota (I library), screened for the ability to break down prebiotic oligosaccharides and polysaccharides (Cecchini et al., 2013).

Based on the functional annotation of the metagenomic DNA insert, we found that 18 of the 43 clones described above contained one or two predicted glycoside transport systems (Table 1) from the SusCD, MFS, ABC, and/or PTS families. The substrates chosen to test the transport ability of these 18 clones correspond to the oligosaccharides used to screen for glycosidase activity via positive selection on agar plates (Cecchini et al., 2013) and to the commercially available oligosaccharides which are components of the polysaccharides used to screen for endo-acting glycoside-hydrolase activities on agar plates (Tasse et al., 2010). However, the enzymatic activity of the fosmid clones might be due to CAZyme-encoding genes which are not part of the same operons (while being present in the same large metagenomic fragment) as the transporter-encoding genes, since PUL boundaries cannot be clearly defined without experimental validation (Moreno-Hagelsieb, 2015). We therefore used the list of known activities for each CAZy family, retrieved from the CAZy database (Lombard et al., 2014; Supplementary Table 1), to enlarge the panel of substrates tested for growth ability, based on all the putative activities of the glycoside hydrolase enzymes (GHs) present in the metagenomic sequences (clones marked with an asterisk in Table 1).

**TABLE 1:** Predicted substrate specificity of glycoside transporters of metagenomic clones, and corresponding transporters and CAZyme families identified in the clone sequences.

Predicted substrate for the transporter	Clone	Clone activity	Screening substrate	Transport system	Glycoside hydrolase enzyme	Library
Fructooligosaccharides	F1	β-fructosidase	FOS	PTS	**GH32**	F
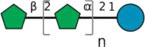	F2	β-fructosidase	FOS	MFS	**GH32**	
	I9	β-fructosidase	FOS	PTS, ABC	**GH32**	I
(0 ≤ n ≤ 3)	I10	β-fructosidase	FOS	ABC	**GH32**	

Xylooligosaccharides	F3	β-xylosidase	XOS	ABC	GH2, **GH3**, GH67	F
	I7	β-xylosidase	XOS	MFS	**GH10**, **GH43**, GH115	I
	I8	β-xylosidase	XOS	MFS, SusC	**GH10**, GH35, **GH43**, GH67, GH115	
	14	β-xylanase	AZCL-xylan	SusCD	**GH5**, **GH10**, **GH43**, GH92	F
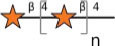 (1 ≤ n ≤ 7)	15	1,4-β-xylanase	AZCL-xylan	SusD	**GH5**, **GH8**, **GH10**, GH31, **GH43**, GH95, GH97, GH115	
	5*	β-glucanase	AZCL-β-glucan	SusD, SusCD	**GH1**, **GH3**, GH16, GH97	F
	9*	β-glucanase	AZCL-β-glucan	SusD	**GH3**, GH7, GH16, GH78	
	10*	β-glucanase	AZCL-β-glucan	SusCD	GH2, **GH3**, **GH5**, GH7, GH94, GH97	
	11*	β-glucanase	AZCL-β-glucan	SusCD	GH2, **GH3**, **GH5**, GH7, GH94, GH97	

Cellobiosyl-cellobiose	3	β-glucanase	AZCL-β-glucan	MFS	GH2, **GH5**	F
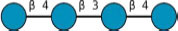	5	β-glucanase	AZCL-β-glucan	SusD, SusCD	**GH1**, **GH3**, **GH16**, GH97	
	9	β-glucanase	AZCL-β-glucan	SusD	**GH3**, **GH7**, **GH16**, GH78	
Glucosyl-cellotriose	10	β-glucanase	AZCL-β-glucan	SusCD	GH2, **GH3**, **GH5**, **GH7**, **GH94**, GH97	
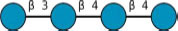	11	β-glucanase	AZCL-β-glucan	SusCD	GH2, **GH3**, **GH5**, **GH7**, **GH94**, GH97	
Cellotriose	14*	1,4-β-xylanase	AZCL-xylan	SusCD	**GH5**, GH10, GH43, GH92	F
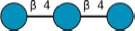	15*	1,4-β-xylanase	AZCL-xylan	SusD	**GH5**, GH8, GH10, GH31, GH43, GH95, GH97, GH115	

Galactosyl-mannobiose	3*	β-glucanase	AZCL-β-glucan	MFS	**GH2**, **GH5**	F
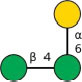	5*	β-glucanase	AZCL-β-glucan	SusD, SusCD	**GH1**, GH3, GH16, GH97	
	10*	β-glucanase	AZCL-β-glucan	SusCD	**GH2**, GH3, **GH5**, GH7, GH94, GH97	
Mannotriose	11*	β-glucanase	AZCL-β-glucan	SusCD	**GH2**, GH3, **GH5**, GH7, GH94, GH97	
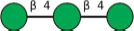	14*	1,4-β-xylanase	AZCL-xylan	SusCD	**GH5**, GH10, GH43, GH92	
Lactulose	15*	1,4-β-xylanase	AZCL-xylan	SusD	**GH5**, GH8, GH10, GH31, GH43, GH95, GH97, GH115	
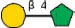	I11	β-galactosidase	Lactulose	MFS/PTS	**GH2**	I
	I12	β-galactosidase	Lactulose	ABC	**GH2**, **GH42**	
	I13	β-galactosidase	Lactulose	PTS	**GH2**, GH77	
	I14	β-galactosidase	Lactulose	ABC, MFS/PTS	**GH2**, GH13	

Lactose	5*	β-glucanase	AZCL-β-glucan	SusD, SusCD	**GH1**, GH3, GH16, GH97	F
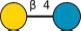						

*Clone activity corresponding to GH activities was screened and validated in previous studies (Tasse et al., 2010; Cecchini et al., 2013). The GH families that might be involved in the hydrolysis of the predicted transporter substrates are shown in bold type. For clones marked with an asterisk, transporter specificity was predicted based on the potential activities of the GHs identified from the metagenomic sequence of the clone. The glycosyl units are represented using the symbol nomenclature for glycans (Varki et al., 2009): glucose in a blue circle, galactose in a yellow circle, mannose in a green circle, fructose in a green pentagon, and xylose in an orange star.*

### Screening for Glycoside Metabolizing Pathways

**FIGURE 1:**
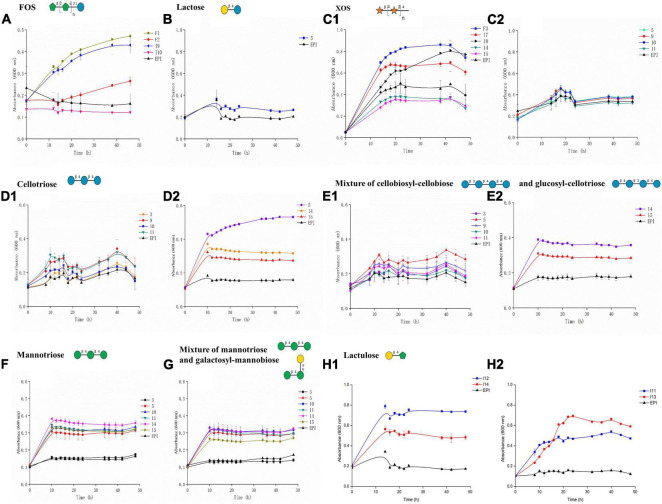
Growth of metagenomic clones on various oligosaccharides used as sole carbon sources: **(A)** FOS, **(B)** lactulose, **(C)** XOS, **(D)** cellotriose, **(E)** mixture of cellobiosyl-cellobiose and glucosyl-cellotriose, **(F)** mannotriose, **(G)** mixture of galactosyl-mannobiose and mannotriose, and **(H)** lactulose. The data represent the averages from biological triplicates, and the scale bars represent the standard deviation (SD).

Utilization of glycosides requires the cooperation of transporters and CAZymes. Previously, we proved that metagenomic gene expression in fosmids is not controlled by promoters flanking the cloning site, but rather, by sequences randomly scattered across the metagenomic DNA insert, which are recognized as promoters by *E. coli* (Tauzin et al., 2016). Therefore, to assess the efficiency of cloning PULs in fosmids to (randomly) co-express at least two genes (encoding a transporter and a glycosidase), and to validate the specificity predictions for the transporters listed in Table 1, growth screening of the 18 metagenomic clones selected for the predicted oligosaccharides was performed in micro-plates. Their growth was characterized by monitoring the optical density value at 600 nm (OD_600 nm_) in M9 medium supplemented with selected oligosaccharides as sole carbon source within 48 h (Figure 1). The *E. coli* host transformed with an empty fosmid (named EPI) was used as a negative control. The increasing value of OD_600 nm_ over time compared with that of EPI provides direct evidence of glycoside utilization by the clone tested.

A total of 44 growth assays were carried out for the 18 selected metagenomic clones, resulting in the identification of 10 hit clones with an OD_600 nm_ value exceeding the value of the negative control EPI by 0.2. Such an increase is significant, since the OD_600 nm_ value of the EPI strain increases by 0.3 after 48 h growth on 0.5% (m/v) glucose. The mean total hit yield was 77.1% when the primary screening for GH activity was based on positive selection on oligosaccharides as sole carbon sources, and 14.3% when it was based on chromogenic assays for screening on polysaccharides (Table 2). Based on sequence analyses, the size of the contigs for each of the 10 hit clones exhibits a wide range, from 13,000 to 43,000 bp, and clone I9 contains two contigs for one single metagenomic DNA insert (Table 3). The clone sequences are mainly assigned to *Firmicutes* and *Bacteroidetes*, the two dominant phyla in the human gut microbiota. The hit clones possess one or two transport systems including ABC, MFS, PTS, and/or SusCD, and from one to six glycoside hydrolases, variously exhibiting the ability to metabolize FOS, XOS, lactulose, or cellotriose. Clone 5, harboring a GH1, was tested on lactose, but was unable to grow on this substrate. Also, 7 clones were tested on mannooligosaccharides or mixed-linked glucooligosaccharides, since their sequences contain a GH that might be involved in the breakdown of these substrates. However, their growth ability was under the threshold fixed in this study to identify the most efficient transporters expressed in *E. coli*. Finally, all the 10 hit clones were able to grow on the same oligosaccharides that had been experimentally tested during the initial positive selection on solid medium (clones F1, F3, I7, I8, I9, I11, I12, I13, and I14) or on oligosaccharides derived from the polysaccharide used for chromogenic assays (clone 5).

**TABLE 2:** Number of hit clones obtained from different libraries and hit yield in growth screening.

	Method for primary screening of GH activity on solid plates		
	Positive selection on oligosaccharides (Cecchini et al., 2013)		Chromogenic assays on polysaccharides (Tasse et al., 2010)
Library	F	I	F
Number of hit clones/number of clones tested in the present study	2/3	7/8	1/7
Hit yield (%)	66.7	87.5	14.3
Mean hit yield (%)	77.1		14.3

**TABLE 3:** Summary of hit clones with the substrate used for growth screening, their contig size and taxonomic assignment at phylum, family or genus.

Substrate	Hit clone	Contig size (bp)	Taxonomic assignment	
			Phylum	Family/Genus
FOS	F1	33,125	Firmicutes	Oscillospiraceae/*Ethanoligenens*
	I9	14,714	Firmicutes	Lachnospiraceae*/Dorea*
		13,700	Firmicutes	Lachnospiraceae/N.A.
XOS	F3	35,645	Firmicutes	Oscillospiraceae*/Subdoligranulum*
	I7	37,812	Bacteroidetes	Prevotellaceae/*Prevotella*
	I8	38,518	Bacteroidetes	Prevotellaceae/*Prevotella*
Cellotriose	5	42,497	Bacteroidetes	Bacteroidaceae/*Bacteroides*
Lactulose	I11	30,347	Firmicutes	Streptococcaceae*/Streptococcus*
	I12	13,317	Firmicutes	Lachnospiraceae/*Roseburia*
	I13	32,036	Firmicutes	Oscillospiraceae*/Faecalibacterium*
	I14	30,347	Firmicutes	Streptococcaceae/*Streptococcus*
Mannotriose and galactosyl-mannobiose	−	−	−	−
Lactose	−	−	−	−

*N.A., not assigned.*

### Identification of Functional Glycoside Transporters

In the metagenomic clones, it is likely that the CAZymes could not be secreted or attached at the cell surface of the recombinant host (Tasse et al., 2010) because of the very low secretion potential of *E. coli* (Kleiner-Grote et al., 2018). The growth of hit clones thus requires functional transporters to import oligosaccharides into cells for further degradation by CAZymes. Therefore, the strategy of using growth assays for screening clones encoding both transporters and CAZymes provides direct experimental evidence of glycoside transporter functionality.

**FIGURE 2:**
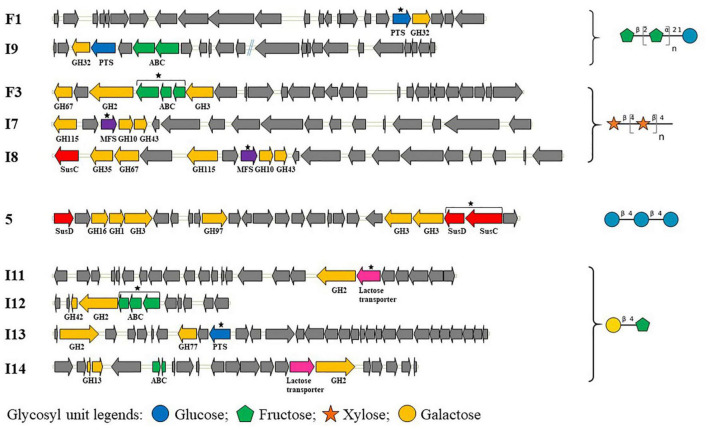
Gene organization of hit clones able to grow on different glycosides. Black stars denote the transporters predicted to be responsible for the clone phenotypes, because they are the only glycoside transport systems detected in the metagenomic PULs or because the SusD component of the SusCD system is missing. The two contigs of clone I9 are separated by blue double lines. Genes are represented by arrows and colored based on their function: genes encoding GHs are shown in yellow, glycoside transporters in blue for PTS, green for ABC, red for SusCD, purple for MFS and pink for “lactose transporter,” and other functions in gray.

The sequences of the 10 hit clones shown in Figure 2 include 14 transport systems belonging to the ABC, PTS, MFS, and/or SusCD families. For six clones (F1, F3, I7, I11, I12, and I13), there is only one putative glycoside transporter in each clone (Figure 2), leading to straightforward identification of the likely specificity of the transporter (given that some genes of unknown function are present in the contigs). Among these six clones, we identified a PTS transporter for FOS (clone F1), and an ABC transport system and an MFS transporter for XOS (clones F3 and I7, respectively). For clones able to grow on lactulose, an ABC transport system and a PTS transporter were identified in clones I12 and I13, respectively, while clone I11 harbors a putative protein named “lactose transporter.” This putative protein includes both a PTS_IIA domain and an MFS domain in the polypeptide chain. For the other four clones (I8, I9, 5, and I14), two transport systems were annotated within the same metagenomic insert (Figure 2). These two transport systems might be working in synergy, completely independently or share redundant function. It is difficult to determine which of the two transporters detected was responsible for the substrate internalization in the growth assays based only on the organization of transporter- and CAZyme-encoding genes. It is indeed impossible to define PUL boundaries in the absence of experimental evidence of operon presence in the native strain from which the metagenomic DNA fragments derive, and because PULs could have been truncated during the construction of the metagenomic library. In particular, clone 5 harbors two SusCD transport systems which could belong to two different PULs based on the distance between them and their genomic environment. One of the PULs lacks the SusC protein, likely due to truncation when constructing the metagenomic library. Thus, the functional transporter in clone 5 can only be the entire SusCD system. In the native strain, it is also possible that the two PULs work in synergy, as demonstrated for several PULs such as the pectin, xylan and N-glycan PULs (Cuskin et al., 2015; Rogowski et al., 2015; Luis et al., 2018). In clone I9, it is impossible at this stage to know whether it is the PTS or the ABC transport system that is responsible for the transport of FOS. Clone I7 is partially redundant with clone I8, sharing 99% nucleotide identity from GH115 to GH43, which covers only one transport system. Clone I8 actually has two transport systems, MFS and SusC, while clone I7 harbors only the MFS transporter. The absence of SusD from clone I8, which could be due to truncation of the PUL when constructing the metagenomic library, suggests that the SusC transport system cannot import any glycosides (Koropatkin et al., 2008; Tauzin et al., 2016; Tamura et al., 2019). Although further investigation is required to confirm this hypothesis, this suggests that the MFS transporter is more likely responsible for the uptake of XOS than the incomplete SusCD transport system in clone I8. Two transport systems are present in clone I14, one annotated as a “lactose transporter,” and an ABC transporter. The “lactose transporter”-encoding gene was clustered with the GH2-encoding gene. The DNA segment including both the GH2 and the lactose transporter showed complete synteny with the corresponding part of sequence I11. This sequence contains just one transport system, shown here to be responsible for the use of lactulose. This implies that the “lactose transporter” might be responsible for the import of lactulose by clone I14 too. Nevertheless, even though the I14 ABC transport system is remote from the “lactose transporter” and the GH2, at this stage we cannot exclude that the ABC might be involved in lactulose utilization too.

## Discussion

### Development of a Screening Method to Identify Glycoside Transporters From Uncultured Bacteria

Glycoside metabolism in bacteria is orchestrated by specific operons involving glycoside-degrading enzymes and transporters, for which the generic PUL appellation can be used (Ndeh and Gilbert, 2018). Here, basing our study on the PUL paradigm, we screened the specificity of glycoside transporters from uncultured bacteria, the sequences of which were previously identified by activity-based functional metagenomics targeting GH activities. We identified 10 metagenomic PULs (three being partially or fully redundant) with glycoside transporter components that are functional in *E. coli*.

In total, 14 transporters of four types (PTS, ABC, MFS, and SusCD) were highlighted from only 44 growth assays, demonstrating the efficiency of this strategy for identifying glycoside transporters with various specificities, structures and transport mechanisms. The present method is highly reproducible, quantitative, and can be performed at medium throughput, screening around 50 clones in 2 days by automatically monitoring growth with a thermostatic microplate reader. This assay requires less than 10 mg of oligosaccharides for each clone, tested in triplicate. It does not require the use of chromogenic, fluorogenic, or radiolabeled substrates. It thus enables easy screening of transporter specificity toward the natural oligosaccharides that bacteria need to take up in nature, preventing positive clones from being missed and avoiding false positives with chemically modified substrates, which have a different structure from natural compounds. Screening for oligosaccharides also provides direct access to transporter function, in contrast to transcriptomic studies which are based on the use of the polymeric substrate before any enzymatic degradation to trigger PUL expression. It also requires 10 times less substrate than the growth assays required for transcriptomic analyses (Martens et al., 2011), which require sufficient cells to be retrieved for RNA extraction and sequencing. And of course, our approach allows for screening for the specificity of transporters from uncultured bacteria.

Nevertheless, there are some limitations in screening for transporter specificity in recombinant *E. coli* cells transformed with PUL-containing fosmids. First, the yield of hit clones producing both a functional glycosidase and a functional transporter is low. As shown in this study, depending on the approach taken in the primary screen targeting GH activity, the hit yield varies between 14 and 77%. Of course, the highest hit yield was obtained when the primary screen was a solid-plate assay based on positive selection on oligosaccharides used as sole carbon sources. Even if the screening principles appear identical in solid medium and in liquid medium, they are in fact slightly different. In solid plate screening campaigns, automates grid several cells of the same clone at a single location on the agar plate. In the GH producing hits, when one of these cells breaks down, it triggers nearby hydrolytic activity. Monosaccharides are then produced in the immediate environment of the other cells in the clonal population, allowing them to grow in turn, and thereby making it easy to visualize cell growth. This principle explains why the hit yield is not 100% after the secondary screening for the transport functions in liquid medium. Screening in liquid medium could be carried out directly in a single stage, without primary screening for GH function. This would require 10 times more substrate for each clone (without replicates), though, and the maximum hit yield would drop to 0.7‰. This value corresponds to the maximum mean probability (4.7‰) of isolating a clone producing an active GH from a library of fosmid clones [8‰ for endo-acting GHs (Tasse et al., 2010) and 1.35‰ for glycosidases (Cecchini et al., 2013) multiplied by that of isolating a functional transporter from GHs producing *E. coli* fosmid clones (14%, as shown in the present study)]. In addition, the probability of producing both a functional glycosidase and a functional transporter from a (meta)genomic PUL cloned into a fosmid is lower than 4.8%, since no hit was obtained from the 21 assays performed based on sequence analysis. This low hit yield is due to several reasons. Firstly, in fosmids, gene expression is spurious as a result of the random presence of sigma factor sequences along the fosmid DNA insert, which are recognized by *E. coli* to trigger gene expression (Tauzin et al., 2016). Secondly, for some multi-domains transporters, several genes of the metagenomic locus have to be concomitantly expressed at similar levels. Finally, to be functional, the recombinant transporters must be properly addressed and folded into the cell membranes of the host strain, and, for some of them, functionally complemented by *E. coli* components, such as TonB for SusCD systems or the phosphoenolpyruvate dependent glycoside phosphorylation machinery for PTSs (Wang et al., 2020).

Screening the transporter function by expressing the genes of PULs in *E. coli* fosmid clones is thus a strategy which requires automated screening facilities. It is also a costly approach, but one for which the cost could be reduced by 10^6^ by using droplet milli/microfluidics, as we recently showed (Dagkesamanskaya et al., 2018). Lastly, by using this technology, the transporter function can be proven only after truncation of the PUL in cases where there are several identified transporter sequences in the cloned DNA fragment (Tauzin et al., 2016), and also in order to exclude a potential role of unknown proteins whose function cannot be predicted in the phenotype detected.

### Major Facilitator Superfamily, ATP-Binding Cassette, Phosphotransferase Systems, and SusCD Transporters From Various Gram-Positive and Gram-Negative Uncultured Bacteria Are Functional in the *E. coli* Membrane

All the metagenomic DNA sequences of the hit clones were assigned to the two main phyla in mammalian gut microbiomes, *Bacteroidetes* and *Firmicutes*. Sequences from bacteria of five different families and eight different genera were identified. However, in any case, it was not possible to annotate the hit sequences to species level, highlighting the functional diversity that remains to be discovered from gut microbiomes by using metagenomics.

*Bacteroidetes* transporters from both the MFS and SusCD families were highlighted in the present study. We previously demonstrated that these two types of membrane proteins can be functional when produced in recombinant *E. coli* cells, whose membrane structure is similar to that of *Bacteroidetes*, both being Gram-negative bacteria (Tauzin et al., 2016). No PTS or ABC systems were identified in these *Bacteroidetes* sequences. This is logical for PTS, which, to our knowledge, have never been reported in *Bacteroidetes*, in contrast to MFS and ABC (Tauzin et al., 2016; Zafar and Saier, 2018) which are described in this phylum, although no *Bacteroidetes* ABC transporter has previously been shown to be involved in carbohydrate transport.

We identified a greater diversity of transporter types from *Firmicutes*, since three PTS, four ABC, and two lactose transporters including one PTS_IIA domain and one MFS domain were highlighted in the Firmicutes’ PULs. MFS, ABC, and PTS systems are all used by native *E. coli* strains for nutrient uptake (Saier, 2000). In addition, several PTS involved in oligosaccharide utilization by Gram-positive bacteria were previously successfully expressed in *E. coli* (Lai and Ingram, 1993; Lai et al., 1997; Wang et al., 2020), despite the difference in cell membrane organization. The localization in *E. coli* of the different components of the transporters identified here from Gram-positive bacteria will need to be explored.

### Non-canonical ATP-Binding Cassette Transporters Are Involved in Oligosaccharide Utilization by Bacteria

Among the ABC transport systems identified, there are two that lack an SBP in clones I9 and I14. Further characterization of this SBP-deficient ABC transporter should shed new light on the mechanism of ABC transporters involved in carbohydrate utilization. With regard to the PTS transport systems, the F1 system lacks the PTS_EIIA domain [the inner carbohydrate-specific domain through which the phosphate group transits to the PTS_EIIB domain and, ultimately, to the oligosaccharide internalized through the transmembrane PTS_EIIC component (Saier, 2015)]. Moreover, the “lactose transporter” identified in clones I11 and I14 for lactulose uptake are original proteins, as the “lactose transporter” contains two domains (a PTS_EIIA and an MFS) belonging to two different types of transporters. The DNA sequences encoding the “lactose transporter” and GH2 of clones I11 and I14 are found near-identical in the genomes of numerous *Streptococcus thermophilus* strains. This demonstrates that the chimeric organization of the transporters identified is not an artifact of read assembly in our metagenomic sequences. The characterization of the role of each component of this I11/I14 original transporter will need to be determined by protein engineering.

## Conclusion

The main purpose of this study was to identify functional transporters from uncultured bacteria. The approach we described in this paper allowed us to identify functional transporters from various families and of different taxonomical origins. We demonstrated the generic application of this strategy, which is compatible with all kinds of known oligosaccharide transporter families described to date, regardless of the Gram status of the bacteria from which they originate. It is now possible to prove transporter translocation function at medium throughput, while other recombinant approaches only target binding ability. We highlighted several gene clusters involved in dietary fiber utilization by gut bacteria, including FOS and lactulose, which are widely used as prebiotics (Bindels et al., 2015). The detailed mechanism and specificity of each of these transporters, especially those with the most novel structure or whose genes are the most abundant in gut microbiomes, will need to be deciphered in future studies.

## Materials and Methods

### Metagenomic Clones

The metagenomic clones used for the present screening were obtained from the F or I metagenomic libraries constructed as previously described (Tasse et al., 2010; Cecchini et al., 2013). Briefly, the F library was derived from a fecal sample collected from a healthy 30-year-old male who followed a pescatarian diet. The individual did not eat any functional food such as prebiotics or probiotics, nor did he receive any antibiotics or other drugs in the 6 months prior to sampling. The I library was derived from a distal ileum sample obtained from a 51-year-old male patient undergoing colonoscopy and surgery for suspected lower colon cancer, after undergoing preparatory cleansing. The metagenomic DNA fragments were cloned into pCC1FOS fosmids and transformed into EPI100 *E. coli* cells (Epicenter Technologies).

### Carbohydrate Substrates

The following commercial prebiotic oligosaccharides and polysaccharides were used for screening: fructooligosaccharides (Actilight-FOS, Beghin Meiji, France), xylooligosaccharides (Iro Taihe International, China), lactulose (TEVA, France), lactose (Sigma), cellobiosyl-cellobiose (Megazyme), glucosyl-cellotriose (Megazyme), cellotriose (Megazyme), galactosyl-mannobiose (Megazyme), and mannotriose (Megazyme). All of them were prepared at 10 mg/mL in sterilized MilliQH_2_O and then filtered using a 0.20 mm Minisart RC4 syringe filter.

### Growth Assays

All the metagenomic clones tested in our study were stored in glycerol stocks stored at −80°C. They were recovered on Luria-Bertani (LB) agar plates supplemented with 12.5 mg/l chloramphenicol. Single colony inoculates were then grown overnight at 37°C in tubes containing 2 mL LB broth supplemented with 12.5 mg/L chloramphenicol, with orbital shaking at 200 rpm. Cultures in 48-well microplates of 500 μL of M9 medium supplemented with 12.5 mg/L chloramphenicol and 0.5% (w/v) oligosaccharides were then started at an initial OD_600 nm_ of 0.05 by inoculating the wells from the overnight precultures. The M9 medium contained 17.4 g/L Na_2_HPO_4_⋅12H_2_O, 3.03 g/L KH_2_PO_4_, 0.51 g/L NaCl, 2.04 g/L NH_4_Cl, 0.49 g/L MgSO_4_, 4.38 mg/L CaCl_2_, 15 mg/L Na_2_EDTA⋅2H_2_O, 4.5 mg/L ZnSO_4_⋅7H_2_O, 0.3 mg/L CoCl_2_⋅6H_2_O, 1 mg/L MnCl_2_⋅4H_2_O, 1 mg/L H_3_BO_3_, 0.4 mg/L Na_2_MoO_4_⋅2H_2_O, 3 mg/L FeSO_4_⋅7H_2_O, 0.3 mg/L CuSO_4_⋅5H_2_O, 0.1 g/L thiamine, and 0.02 g/L leucine. Cell growth was monitored at 37°C, with orbital shaking at 600 rpm. The optical density of the cultures was measured at 600 nm over 48 h, using a plate reader (Infinite M200pro, 593 TECAN).

### Bioinformatic Analysis

The functional annotation of the metagenomic sequences was retrieved from previous studies (Tasse et al., 2010; Cecchini et al., 2013). Functional annotation of the transporter-encoding genes was confirmed by analyzing the conserved domains of the putative proteins against the NCBI Conserved Domains Database using BLAST (the NCBI Basic Local Alignment Search Tool), with an *E*-value threshold of 0.01 (Marchler-Bauer et al., 2017). When an incomplete transport system was detected (missing the neighboring SusD gene, or one of the components of canonical PTS and ABC transporters), we checked for the presence of the complementary component by analyzing the conserved domains of the two proteins encoded by the genes before or after the main transporter-encoding gene. Sequence identity between metagenomic DNA inserts was analyzed using BLAST (Boratyn et al., 2013). Taxonomic assignment of the contig sequences was performed using PhyloPythiaS, model Generic 2013–800 Genera^1^ (Patil et al., 2012).

## Data Availability Statement

The datasets presented in this study can be found in online repositories. The names of the repository/repositories and accession number(s) can be found below: https://www.ncbi.nlm.nih.gov/genbank/, GU942948.1; www.ncbi.nlm.nih.gov/genbank/, HE663537; www.ncbi.nlm.nih.gov/genbank/, HE717013; www.ncbi.nlm.nih.gov/genbank/, HE717008; www.ncbi.nlm.nih.gov/genbank/, HE717009; www.ncbi.nlm.nih. gov/genbank/, HE717006; www.ncbi.nlm.nih.gov/genbank/, HE717007; https://www.ncbi.nlm.nih.gov/genbank/, HE717018; www.ncbi.nlm.nih.gov/genbank/, HE717019; www.ncbi.nlm.nih. gov/genbank/, HE717014; and www.ncbi.nlm.nih.gov/genbank/, HE717020.

## Author Contributions

GP-V: conceptualization, writing—review and editing, and resources. AT, ZW, and EL: methodology and investigation. ZW and AT: writing—original draft. GP-V, AT, and ZW: funding acquisition. GP-V and AT: supervision. All authors contributed to the article and approved the submitted version.

## Conflict of Interest

The authors declare that the research was conducted in the absence of any commercial or financial relationships that could be construed as a potential conflict of interest.

## Publisher’s Note

All claims expressed in this article are solely those of the authors and do not necessarily represent those of their affiliated organizations, or those of the publisher, the editors and the reviewers. Any product that may be evaluated in this article, or claim that may be made by its manufacturer, is not guaranteed or endorsed by the publisher.

## Footnotes

1
http://binning.bioinf.mpi-inf.mpg.de/

## Supplementary Material

Supplementary Table 1 Known enzymatic activity of glycoside hydrolase (GH) families in the selected clones based on the CAZy database and the substrates selected (based on activities in bold) for growth tests.
