# Supplementary material for: Identification of Glycoside Transporters From the Human Gut Microbiome
Source: Front Microbiol. 2022 Mar 25;13:816462. doi: 10.3389/fmicb.2022.816462 (PMC8990778; doi:10.3389/fmicb.2022.816462)
Supplement: Supplementary Table 1 — Known enzymatic activity of glycoside hydrolase (GH) families in the selected clones based on the CAZy database and the substrates selected (based on activities in bold) for growth tests. [file Table_1.DOCX]

| **GH families** | **Known activities listed in the CAZy database** | **Selected substrates tested for growth screening** |
| --- | --- | --- |
| GH1 | **β-glucosidase** **(EC 3.2.1.21)**; **β-galactosidase** **(EC 3.2.1.23)**; **β-mannosidase** **(EC 3.2.1.25)**; β-glucuronidase (EC 3.2.1.31); **β-xylosidase** **(EC 3.2.1.37)**; β-D-fucosidase (EC [3.2.1.38](http://www.enzyme-database.org/query.php?ec=3.2.1.38)); phlorizin hydrolase (EC [3.2.1.62](http://www.enzyme-database.org/query.php?ec=3.2.1.62)); exo-β-1,4-glucanase (EC 3.2.1.74); 6-phospho-β-galactosidase (EC 3.2.1.85); 6-phospho-β-glucosidase (EC 3.2.1.86); strictosidine β-glucosidase (EC 3.2.1.105); lactase (EC 3.2.1.108); amygdalin β-glucosidase (EC 3.2.1.117); prunasin β-glucosidase (EC [3.2.1.118](http://www.enzyme-database.org/query.php?ec=3.2.1.118)); vicianin hydrolase (EC 3.2.1.119); raucaffricine β-glucosidase (EC 3.2.1.125); thioglucosidase (EC 3.2.1.147); β-primeverosidase (EC 3.2.1.149); isoflavonoid 7-O-β-apiosyl-β-glucosidase (EC 3.2.1.161); ABA-specific β-glucosidase (EC 3.2.1.175); DIMBOA β-glucosidase (EC 3.2.1.182); β-glycosidase (EC [3.2.1.-](http://www.enzyme-database.org/query.php?ec=3.2.1.*)); hydroxyisourate hydrolase (EC [3.-.-.-](http://www.enzyme-database.org/query.php?ec=3.*.*.*)) | XOS;  Galactosyl-mannobiose and mannotriose;  Cellobiosyl-cellobiose, glucosyl-cellotriose, and cellotriose;  Lactose |
| GH2 | **β-galactosidase (EC 3.2.1.23)**; **β-mannosidase (EC 3.2.1.25)**; β-glucuronidase (EC 3.2.1.31); α-L-arabinofuranosidase (EC 3.2.1.55); mannosylglycoprotein endo-β-mannosidase (EC 3.2.1.152); exo-β-glucosaminidase (EC 3.2.1.165); α-L-arabinopyranosidase (EC 3.2.1.-); β-galacturonidase (EC 3.2.1.-); β-xylosidase (EC 3.2.1.37); β-D-galactofuranosidase (EC 3.2.1.146) | Galactosyl-mannobiose and mannotriose;  Lactulose |
| GH3 | **β-glucosidase (EC 3.2.1.21)**; **xylan 1,4-β-xylosidase** **(EC 3.2.1.37)**; β-glucosylceramidase (EC 3.2.1.45); β-N-acetylhexosaminidase (EC 3.2.1.52); α-L-arabinofuranosidase (EC 3.2.1.55); glucan 1,3-β-glucosidase (EC 3.2.1.58); glucan 1,4-β-glucosidase (EC 3.2.1.74); isoprimeverose-producing oligoxyloglucan hydrolase (EC 3.2.1.120); coniferin β-glucosidase (EC 3.2.1.126); exo-1,3-1,4-glucanase (EC 3.2.1.-); β-N-acetylglucosaminide phosphorylases (EC 2.4.1.-); β-1,2-glucosidase (EC 3.2.1.-); β-1,3-glucosidase (EC 3.2.1.-); xyloglucan-specific exo-β-1,4-glucanase/exo-xyloglucanase (EC 3.2.1.155) | Xylosaccharides;  Cellobiosyl-cellobiose, glucosyl-cellotriose, and cellotriose (β-glucosidase); |
| GH5 | endo-β-1,4-glucanase/cellulase (EC 3.2.1.4); **endo-β-1,4-xylanase (EC 3.2.1.8)**; **β-glucosidase (EC 3.2.1.21)**; **β-mannosidase (EC 3.2.1.25)**; β-glucosylceramidase (EC 3.2.1.45); glucan β-1,3-glucosidase (EC 3.2.1.58); exo-β-1,4-glucanase/cellodextrinase (EC 3.2.1.74); glucan endo-1,6-β-glucosidase (EC 3.2.1.75); mannan endo-β-1,4-mannosidase (EC 3.2.1.78); cellulose β-1,4-cellobiosidase (EC 3.2.1.91); steryl β-glucosidase (EC 3.2.1.104); endoglycoceramidase (EC 3.2.1.123); chitosanase (EC 3.2.1.132); β-primeverosidase (EC 3.2.1.149); xyloglucan-specific endo-β-1,4-glucanase (EC 3.2.1.151); endo-β-1,6-galactanase (EC 3.2.1.164); β-1,3-mannanase (EC 3.2.1.-); arabinoxylan-specific endo-β-1,4-xylanase (EC 3.2.1.-); mannan transglycosylase (EC 2.4.1.-); lichenase/endo-β-1,3-1,4-glucanase (EC 3.2.1.73); β-glycosidase (EC 3.2.1.-); endo-β-1,3-glucanase/laminarinase (EC 3.2.1.39); β-N-acetylhexosaminidase (EC 3.2.1.52); chitosanase (EC 3.2.1.132); β-D-galactofuranosidase (EC 3.2.1.146); β-galactosylceramidase (EC 3.2.1.46) | XOS;  Cellobiosyl-cellobiose, glucosyl-cellotriose, and cellotriose;  Galactosyl-mannobiose and mannotriose |
| GH7 | endo-β-1,4-glucanase (EC 3.2.1.4); **reducing end-acting cellobiohydrolase (EC 3.2.1.176)**; chitosanase (EC 3.2.1.132); **endo-β-1,3-1,4-glucanase (EC 3.2.1.73)** | Cellobiosyl-cellobiose, glucosyl-cellotriose, and cellotriose |
| GH8 | Family chitosanase (EC 3.2.1.132); cellulase (EC 3.2.1.4); licheninase (EC 3.2.1.73); **endo-1,4-β-xylanase (EC 3.2.1.8);** **reducing end xylose-releasing exo-oligoxylanase (EC 3.2.1.156)** | XOS |
| GH10 | **endo-1,4-β-xylanase (EC 3.2.1.8)**; endo-1,3-β-xylanase (EC 3.2.1.32); tomatinase (EC 3.2.1.-); xylan endotransglycosylase (EC 2.4.2.-); **endo-β-1,4-glucanase (EC 3.2.1.4)** | XOS;  Cellobiosyl-cellobiose, glucosyl-cellotriose, and cellotriose |
| GH16 | xyloglucan:xyloglucosyltransferase (EC 2.4.1.207); keratan-sulfate endo-1,4-β-galactosidase (EC 3.2.1.103); endo-1,3-β-glucanase/laminarinase (EC 3.2.1.39); **endo-1,3(4)-β-glucanase (EC 3.2.1.6)**; licheninase (EC 3.2.1.73); β-agarase (EC 3.2.1.81); κ-carrageenase (EC 3.2.1.83); xyloglucanase (EC 3.2.1.151); endo-β-1,3-galactanase (EC 3.2.1.181); [retaining] β-porphyranase (EC 3.2.1.178); hyaluronidase (EC 3.2.1.35); endo-β-1,4-galactosidase (EC 3.2.1.-); chitin β-1,6-glucanosyltransferase (EC 2.4.1.-); β-transglycosidase (EC 2.4.1.-); **β-glycosidase (EC 3.2.1.-)**; endo-β-1,3-galactanase (EC 3.2.1.181); β-carrageenase (EC 3.2.1.-) | Cellobiosyl-cellobiose, glucosyl-cellotriose, and cellotriose |
| GH32 | invertase (EC 3.2.1.26); endo-inulinase (EC 3.2.1.7); β-2,6-fructan 6-levanbiohydrolase (EC 3.2.1.64); endo-levanase (EC 3.2.1.65); exo-inulinase (EC 3.2.1.80); **fructan β-(2,1)-fructosidase/1-exohydrolase (EC 3.2.1.153)**; fructan β-(2,6)-fructosidase/6-exohydrolase (EC 3.2.1.154); sucrose:sucrose 1-fructosyltransferase (EC 2.4.1.99); fructan:fructan 1-fructosyltransferase (EC 2.4.1.100); sucrose:fructan 6-fructosyltransferase (EC 2.4.1.10); fructan:fructan 6G-fructosyltransferase (EC 2.4.1.243); levan fructosyltransferase (EC 2.4.1.-); [retaining] sucrose:sucrose 6-fructosyltransferase (6-SST) (EC 2.4.1.-); cycloinulo-oligosaccharide fructanotransferase (EC 2.4.1.-) | FOS |
| GH42 | **β-galactosidase (EC 3.2.1.23)**; α-L-arabinopyranosidase (EC 3.2.1.-) | Lactulose |
| GH43 | **β-xylosidase (EC 3.2.1.37)**; α-L-arabinofuranosidase (EC 3.2.1.55); xylanase (EC 3.2.1.8); α-1,2-L-arabinofuranosidase (EC 3.2.1.-); exo-α-1,5-L-arabinofuranosidase (EC 3.2.1.-); [inverting] exo-α-1,5-L-arabinanase (EC 3.2.1.-); β-1,3-xylosidase (EC 3.2.1.-); [inverting] exo-α-1,5-L-arabinanase (EC 3.2.1.-); [inverting] endo-α-1,5-L-arabinanase (EC 3.2.1.99); exo-β-1,3-galactanase (EC 3.2.1.145); β-D-galactofuranosidase (EC 3.2.1.146) | XOS |
| GH94 | **cellobiose phosphorylase (EC 2.4.1.20)**; laminaribiose phosphorylase (EC 2.4.1.31); **cellodextrin phosphorylase (EC 2.4.1.49)**; chitobiose phosphorylase (EC 2.4.1.-); cyclic β-1,2-glucan synthase (EC 2.4.1.-); cellobionic acid phosphorylase (EC 2.4.1.321); β-1,2-oligoglucan phosphorylase (EC 2.4.1.-) | Cellobiosyl-cellobiose, glucosyl-cellotriose, and cellotriose |
